# Pig fatness in relation to *FASN* and *INSIG2* genes polymorphism and their transcript level

**DOI:** 10.1007/s11033-016-3969-z

**Published:** 2016-03-10

**Authors:** Maria Grzes, Slawomir Sadkowski, Katarzyna Rzewuska, Maciej Szydlowski, Marek Switonski

**Affiliations:** Department of Genetics and Animal Breeding, Poznan University of Life Sciences, Wolynska 33, 60-637 Poznan, Poland

**Keywords:** Pig, Adipose tissue, Fatness, Fatty acids, *FASN*, *INSIG2*

## Abstract

Fat content and fatty acid (FA) profile influence meat quality in pigs. These parameters are important for consumers due to their preferences for healthy, high quality meat. The aim of this study was searching for polymorphisms and transcript levels of two positional and functional candidate genes, *FASN* and *INSIG2*, encoding proteins which take part in lipid metabolism. The molecular findings were analyzed in relation to fatness traits. Pigs of four commercial breeds were included: Polish Landrace (PL), Polish Large White (PLW), Duroc and Pietrain. DNA sequencing, 5′RACE technique and real time PCR and association analysis were applied. In total, 20 polymorphisms in 5′-flanking, 5′UTR and 3′UTR regions of *FASN* (12 novel polymorphisms) and *INSIG2* (seven novel ones and one known) genes were found. Association study with fatness traits (PL n = 225, PLW n = 179) revealed that four polymorphisms (c.-2908G>A, c.-2335C>T, c.*42_43insCCCCA and c.*264A>G) of the *FASN* gene were associated with back fat thickness in PL and PLW. Since the polymorphisms were identified in regulatory sequences of the both genes also their transcript levels were studied in PLW (n = 23), PL (n = 22), Pietrain (n = 17) and Duroc (n = 23). The *INSIG2* transcript level was positively correlated with monounsaturated FA contents in the *longissimus thoracis et lumborum* muscle. Several correlations were also found between three polymorphisms (c.*264A>G and c.-2335C>T in *FASN*, and c.-5527C>G in *INSIG2*) and the FA content. Our study showed that the *FASN* gene is a promising marker for subcutaneous fat tissue accumulation, while *INSIG2* is a promising marker for FA composition.

**Electronic supplementary material:**

The online version of this article (doi:10.1007/s11033-016-3969-z) contains supplementary material, which is available to authorized users.

## Introduction

Fat tissue deposition and fatty acid (FA) composition are important traits influencing pork quality and its dietetic value. Although not much is known on genes or molecular mechanisms underlying the phenotypic variation of fat deposition in different breeds, the contribution of genetic factors to these traits is relatively high, as the heritability coefficient for such traits as BFT (back fat thickness) or IMF (intramuscular fat content) as well as FA composition oscillates around 0.5 [1]. Thus, their improvement by selection is required for at least two reasons. Firstly, nowadays consumers prefer lean meat; however, a 2.5–3 % IMF content is desired as it confers marbling, which in turn is essential for meat tenderness and juiciness [2]. Secondly, FA composition determines the dietetic value of meat and thus has an impact on consumer health [3, 4].

Candidate genes for porcine fat tissue accumulation and FA composition include *FASN* and *INSIG2*, which expression products are involved in lipid metabolism. The former gene encodes FA synthase, an important enzyme that catalyses the biosynthesis of saturated fatty acids (SFA), mainly palmitic acid. The *FASN* gene was mapped to SSC12p1.5, nearby QTLs for BFT and the back fat FA composition [5]. In turn, the *INSIG2* gene encodes a protein which participates in sterol-dependent HMG-CoA reductase degradation and the regulation of SREBP activation [6]. The SREBP transcriptional factor regulates expression of over 30 genes involved in cholesterol and FAs synthesis. Interestingly, one of these genes is *FASN* [7]. Chromosomal localization on SSC15q12, in the vicinity of QTLs for back and abdominal fat thickness, suggests that *INSIG2* is also a positional candidate gene for fatness traits [8].

Since polymorphisms of gene regulatory regions (promoter, 5′UTR and 3′UTR) may affect their transcript level and in consequence may exhibit an association with fat tissue accumulation and FA composition, we aimed to: (1) search for polymorphic variants in the 5′-flanking as well as 5′- and 3′UTR of the porcine *FASN* and *INSIG2* genes, (2) analyze the association of the identified polymorphisms with pig fatness traits, and (3) study the transcript levels of both genes in the *longissimus thoracis et lumborum* (LTL) and subcutaneous fat (SC) with regard to their correlation with adipose tissue accumulation and FA content.

## Materials and methods

### Animals

A total of 85 unrelated gilts, representing four breeds: the Polish Large White (PLW, n = 23), Polish Landrace (PL, n = 22), Pietrain (n = 17) and Duroc (n = 23), were analyzed in order to search for polymorphism and to determine transcript levels of the *FASN* and *INSIG2* genes. Furthermore, for these animals data concerning FA content in LTL and SC, measured by gas chromatography and reported elsewhere [9], were used. The associations between selected polymorphisms and production traits were calculated for PLW (n = 179) and PL (n = 225) gilts. All animals were kept under identical conditions in a local Pig Testing Station (Pawlowice, Poland), fed ad libitum, slaughtered at 100 kg of body weight and dissected. The study was approved by the Local Ethical Commission for Research on Animals in Poznan (Poland).

### DNA sequencing and genotyping

Genomic DNA was isolated from blood samples using the Blood Mini Isolation Kit (A&A Biotechnology). In total 8 primer pairs, 4 for each gene, were designed in the Primer3 tool (http://bioinfo.ut.ee/primer3-0.4.0/). Primer sequences, their annealing temperatures and amplicon lengths as well as localizations are listed in Supplementary Table 1. To screen for polymorphisms direct sequencing was applied. Amplicons, after exonuclease and alkaline phosphatase treatment, were sequenced with the use of the BigDay Terminator Sequencing Kit (Applied Biosystems), followed by purification on a Sephadex G-50 (Sigma Aldrich) and analysis by capillary electrophoresis on a 3130 Genetic Analyzer (Applied Biosystems). Genotyping for association analysis was performed by direct sequencing (*FASN*, c.*42_43insCCCCA and *INSIG2*, c.*423G>A) or by RFLP with the use of the following endonucleases: *Alw*NI (*FASN*, c.-2908G>A), *Sma*I (*FASN*, c.-2335C>T), *Bsr*BI (*FASN*, c.*264A>G), *Hae*III (*INSIG2*, c.-5527C>G). The Haploview software (http://www.broadinstitute.org/scientific-community/science/programs/medical-and-population-genetics/haploview/haploview) was used to estimate haplotype frequencies, TESS (http://www.cbil.upenn.edu/cgi-bin/tess/tess) and P-Match 1.0 Public (http://www.gene-regulation.com/cgi-bin/pub/programs/patch/bin/patch.cgi) softwares were applied to search for potential transcription binding sites in the 5′-flanking regions and EMBOSS Cpglot (http://www.ebi.ac.uk/Tools/seqstats/emboss_cpgplot/) was used in prediction of CpG islands. Polymorphisms within 3′UTR were analyzed with TargetScan (http://www.targetscan.org/) in order to determine whether they are localized within a potential target sequence for miRNA.

### Transcript level

To perform the amplification of cDNA 5′ ends a FirstChoice RLM-RACE Kit (Ambion) was used, according to the manufacturer’s protocol. RNA was isolated from SC of PL pigs. Primers used for 5′RACE are listed in Supplementary Table 1.

Total RNA was extracted from the LTL and SC using the TriPure Isolation Reagent (Roche Diagnostics). The RNA quantity and quality were assessed in a NanoDrop spectrophotometer (Thermo Scientific). Subsequently to reverse transcription of approximately 2.5 µg RNA with random hexamers and oligodT (Roche), the real-time PCR analysis was performed in duplicate on the LightCycler2.0 analyzer (Roche). For fluorescence monitoring SYBR Green I (Roche) was used. The specificity of the amplified fragments was confirmed based on the melting curve and product length analysis. The standard curve for each gene (*FASN* and *INSIG2*, as well as reference genes: *ACTB* and *PPIA*) was designed as a series of tenfold dilutions of the purified PCR product. In each real-time PCR analysis standards of studied and reference genes were included. The relative abundance of *FASN* and *INSIG2* transcripts was normalized to a geometric mean of the two reference genes (*ACTB* and *PPIA*), as it was proposed by Vandesompele et al. [10]. All primer pairs (Supplementary Table 1) for this reaction covered 2 neighboring exons, in order to facilitate detection of potential genomic DNA contamination.

### Statistics analysis

The association analysis between *FASN* and *INSIG*2 gene polymorphisms and fatness traits was conducted with a linear mixed model for each breed separately. The statistical model was as follows:

$$y_{ijkl} = \mu + s_{i} + g_{j} + r_{k} + \beta_{1xl} + \beta_{2zl} + e_{ijkl}$$

where *y*_*ijkl*_ is the trait observed for gilt *ijkl*, *µ* is the overall mean; *s*_*i*_ is the random effect of sire; *g*_*j*_ is the fixed effect of polymorphism under study; *r*_*k*_ is the fixed effect of genotype at the *RYR1* locus (two levels: CC and CT at position 1843); *β*_1_ and *β*_2_ are fixed coefficients of regression, *x*_*k*_ and *z*_*k*_ are the age at slaughter and right half-carcass weight, respectively; and *e*_*ijkl*_ is the residual effect.

Due to the low frequencies of some genotypes, namely: TT (c.-2335C>T) in PLW and PL, AA (c.-2908G>A) in PL, GG (c.-5527C>G) in PL and AA (c.*423G>A) in PLW, they were excluded from genotype-phenotype association studies.

In case of *FASN* gene we also considered an association between fatness traits and haplotypes (c.-2908G>A and c.-2335CT for PL; c.-2908G>A, c.-2335CT and c.*42_43insCCCCA for PLW). We regressed phenotype on haplotype content (0, 1 and 2). When for a gilt the haplotypes could not be fully determined we selected variants with highest population frequencies as estimated by HaploView program. Additive effects of haplotypes were estimated simultaneously by imposing a restriction for their sum to be 0. The statistical model was similar to that described above, except that the effect of genotype was replaced by covariates for the haplotype contents.

Analyses were performed for 12 fatness traits (BFT measured at seven points, IMF, abdominal fat weight, percentage of lean meat in carcass, back fat of ham with skin weight, back fat of loin with skin weight). All statistical calculations were performed using the R software v3.2.0 (R Development Core Team).

Correlations between *FASN* and *INSIG2* transcript levels and FA contents as well as fatness traits were calculated. Due to the skewness of transcript levels the logarithm transformation was applied, followed by the outlier removal (values 2 SD above or below the mean trait value were excluded). To account for the breed effect the correlation was calculated from residuals after application of a fixed linear model with the breed effect included.

The *U* Mann–Whitney and Kruskal–Wallis tests were applied to compare mean levels of transcripts and FAs depending on the polymorphic variants.

## Results and discussion

### *FASN* and *INSIG2* polymorphisms

**Fig. 1:**
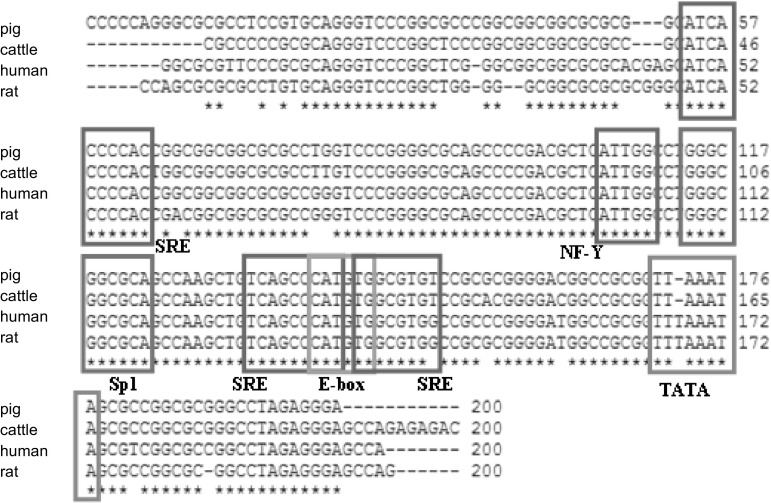
Comparison of 200 bp of the *FASN* gene promoter sequence in four species

Altogether 2127 bp of the *FASN* gene, including 1730 bp of the 5′-flanking sequence and 5′UTR, and 397 bp of 3′UTR were obtained. Using the 5′RACE technique we identified the transcription start site (TSS), expressed in SC, as well as additional 85 bp at the 5′ end of the transcript compared to the sequence deposited in the NCBI GenBank (NM_001099930). In the obtained sequence upstream of TSS all characteristic motifs (TATA-box, SRE/E-box, Sp1-binding GC box, NF-Y binding box) for the *FASN* gene promoter were present (Fig. 1) [11]. Comparison of this sequence (200 bp above TSS) revealed a high level of its similarity (around 90 %) between four species: pig, human, rat and cattle.

Altogether, 12 novel polymorphic sites in the *FASN* gene were found, including 10 in the 5′-flanking region (c.-3220C>T, c.-3023C>T, c.-2943A>C, c.-2908G>A, c.-2863G>A, c.-2649T>C, c.-2631G>T, c.-2392T>G, c.-2335C>T and c.-2333_2334insG) and 2 in 3′UTR (c.*42_43insCCCCA and c.*264A>G). Among them one (c.-3023C>T) was absent in Duroc pigs, whereas the indel (c.*42_43insCCCCA) was present in PLW only (Supplementary Table 2). To our knowledge in the pig *FASN* gene other 10 SNPs, located within the coding sequence (c.196C>T, c.265C>T, c.508T>C, c.826C>T, c.1254A>G, c.2236G>T, c.3082G>A, c.3189T>C, c.5008C>T and c.6545A>C), were reported earlier and three of them altered the amino acids sequence: c.1254A>G (Arg>Gln), c.3189T>C (Thr>Ile), c.6545A>C (His->Asn) [12]. Moreover, they co-segregated as two haplotypes (c.[1254A; 3189T; 6545A] and c.[1254G; 3189C; 6545C]) in the Landrace breed. In 2014 Molnar et al. found in Mangalica and Duroc breeds another two missense SNPs in this gene [13]. One of them was located in exon 9 (G>A, position 1,028,766) and caused amino acid substitution (R443Q), while the second one (C>T in exon21, position 1,025,096) resulted in a T1088I change.

The in silico analysis of the detected polymorphisms in the 5′flanking sequence indicated that some of them (c.-2335C>T, c.-2392T>G, c.-2649T>C, c.-2908G>A, c.-2943A>C and c.-3220C>T) may affect the binding probability of the transcription factors. For example, c.-2335C>T polymorphism occurs within a consensus sequence for the Sp1 transcription factor, while the c.-2943A>C polymorphism is found within a target sequence for the NF-1 transcription factor. Interestingly, all identified polymorphisms in the 5′-flanking region are located within a potential CpG island. On the other hand, the two polymorphic sites in 3′UTR occurred outside potential target sequences for microRNA molecules.

Sequence analysis of the 2462 bp of the *INSIG2* gene, comprising 1270 bp of the 5′-flanking region and 5′UTR, as well as 1192 bp of 3′UTR, revealed 8 polymorphisms. They were found in the 5′-flanking region (c.-5616G>T, c.-5603T>C, c.-5527C>G), 5′UTR (c.-5271G>A) and 3′UTR (c.*423G>A, c.*463C>T, c.*725T>C, c.*793A>C). Among them three (c.-5616G>T, c.-5603T>C and c.-5271G>A) were specific to the Duroc breed, while in the lean Pietrain breed only one SNP (c.*423G>A) was observed (Supplementary Table 2). One SNP (c.-5616G>T) and another 4 polymorphic sites in the promoter and 3′UTR (c.-212-669T>C, c.-212-84G>C, c.*121_122delGT and c.*442A>G) were previously reported in the Ph.D. thesis of Pertek [14]. These four polymorphisms were not found in our study, possibly due to a low minor allele frequency (MAF) or their specificity to the Mangalica breed, since they were detected in Mangalica x Pietrain crossbred pigs.

Two SNPs, c.-5603T>C and c.-5527C>G, alter the putative transcription factor binding sites. The first SNP is located within the sequences recognized by NF-1 and c-Ets-1, whereas the other—within sequences for the AP-1 and Sp1 transcription factors. Similarly to *FASN* gene polymorphisms, none of the *INSIG2* polymorphic sites affected the conserved target sequences for microRNA molecules.

### Associations between *FASN* and *INSIG2* polymorphisms and fatness traits

On the basis of two criteria, namely location within the potential regulatory element sequence and the relatively even distribution of alleles, 4 polymorphisms in the *FASN* gene (c.-2335C>T, c.-2908G>A, c.*264A>G for PL and PLW; and c.*42_43insCCCCA for PLW only) and 2 SNPs in the *INSIG2* gene (c.-5527C>G for PL only and c.*423G>A for PLW only) were selected for the association studies.

The following genotype frequencies at the *FASN* locus were calculated: (1) at c.-2335C>T: 0.57 (CC), 0.39 (CT), 0.04 (TT) in PL, and 0.73, 0.25, 0.02 in PLW; (2) at c.-2908G>A: 0.44 (GG), 0.50 (GA), 0.06 (AA) in PL, and 0.29, 0.53, 0.18 in PLW; (3) at c.*264A>G: 0.32 (AA), 0.60 (AG), 0.08 (GG) in PL, and 0.28, 0.50, 0.22 in PLW, and (4) at c.*42_43insCCCCA: 0.84 (del/del) and 0.16 (ins/del) in PLW. The Chi square test revealed deviations from the Hardy–Weinberg proportion in PL for c.-2908G>A (*FASN*) and c.*264A>G (*FASN*).

With regard to *INSIG2* polymorphism we observed a distinct differences between PL and PLW breeds. At c.-5527C>G site a quite wide distribution of polymorphic variants was observed in PL and the genotype frequencies were as follows: CC (0.59), CG (0.37) and GG (0.04), while in PLW this site was almost monomorphic. A reverse situation was observed at c.*423G>A, which was polymorphic in PLW—genotype frequencies were: 0.70 (GG), 0.26 (GA) and 0.04 (AA), but almost monomorphic in PL. The association analysis did not reveal significant (p > 0.05) associations between the analyzed polymorphisms and fatness traits.

The association analyses revealed a number of statistically significant multiple associations with fatness traits for four polymorphisms in the *FASN* gene (Table 1). Interestingly, all analyzed polymorphisms were associated with adipose tissue accumulation, mainly with back fat thickness. For instance, in the GG homozygotes (c.*264A>G in *FASN* gene) higher values of BFT over the back and BFT at *sacrum* point III were found. In turn, deletion of c.*42_43insCCCCA (*FASN*) was associated with a lower BFT over back and a lower back fat of ham with skin weight. We observed a high correlation between c.-2908G>A and c.*264A>G (linkage disequilibrium R^2^ = 0.98), therefore we believe these two markers capture the same phenotypic effect on BFT. A synonymous mutation in exon 4 (c.265C>T) was tested by Renaville et al. [15]. No effect on BFT was observed, but an association with weight loss during salting in four hybrids was found. The search for an association between *FASN* gene polymorphisms and pork fatness traits was also carried out by Kim et al. [16]. They reported an association between c.6545A>C and IMF content in Korean native x Yorkshire crossbred pigs. Taking into consideration the detected associations, as well as the chromosomal localization of the *FASN* on SSC12 nearby QTLs for BFT and the back fat FA composition, it might be suggested that a functional polymorphism, exerting a significant phenotypic impact on BFT, may occurs within or in the vicinity of the *FASN* gene.

**Table 1:** Associations between *FASN* polymorphisms and pig fatness traits—the marginal means with standard errors and p value for the polymorphism effect

Polymorphism	Breed (n)	Trait	Mean ± SE (n)			p value
			11^b^	12^c^	22^d^	
c.-2908G>A	PLW (179)	BFT at *sacrum* point III (cm)	1.44 ± 0.08 (33)	1.23 ± 0.06 (94)	1.30 ± 0.07 (52)	0.016
c.-2335C>T	PLW (179)	BFT at *sacrum* point III (cm)	1.34 ± 0.07 (133)	1.21 ± 0.08 (46)	Not analyzed^a^	0.047
		Back fat of loin with skin (kg)	1.68 ± 0.05 (133)	1.57 ± 0.06 (46)	Not analyzed^a^	0.040
c.*42_43insCCCCA	PLW (191)	BFT over back (cm)	1.22 ± 0.05 (161)	1.37 ± 0.08 (30)	Not analyzed^a^	0.026
		Back fat of ham with skin (kg)	1.50 ± 0.04 (161)	1.58 ± 0.05 (30)	Not analyzed^a^	0.049
c.*264A>G	PL (225)	BFT over back (cm)	1.31 ± 0.05 (69)	1.31 ± 0.04 (139)	1.65 ± 0.09 (17)	0.0005
	PLW (187)	BFT at *sacrum* point III (cm)	1.30 ± 0.07 (52)	1.23 ± 0.06 (94)	1.42 ± 0.08 (41)	0.025

^a^Not analyzed due to a low number of animals with the given homozygous genotype

^b^Homozygotes for a wild allele (according to the reference genome sequence)

^c^Heterozygotes

^d^Homozygotes for the detected polymorphic variant

In the case of the porcine *INSIG2* the association between the heterozygous genotype at c.*121_122delGT and a higher BFT in Mangalica x Pietrain crossbred pigs was mentioned by Pertek [14]. In our study we found no significant (p > 0.05) associations between the analyzed *INSIG2* gene polymorphisms and fatness traits, thus only above mentioned four *FASN* gene polymorphisms may be used as molecular markers for pig BFT.

### Transcript levels of *FASN* and *INSIG2* genes

**Fig. 2:**
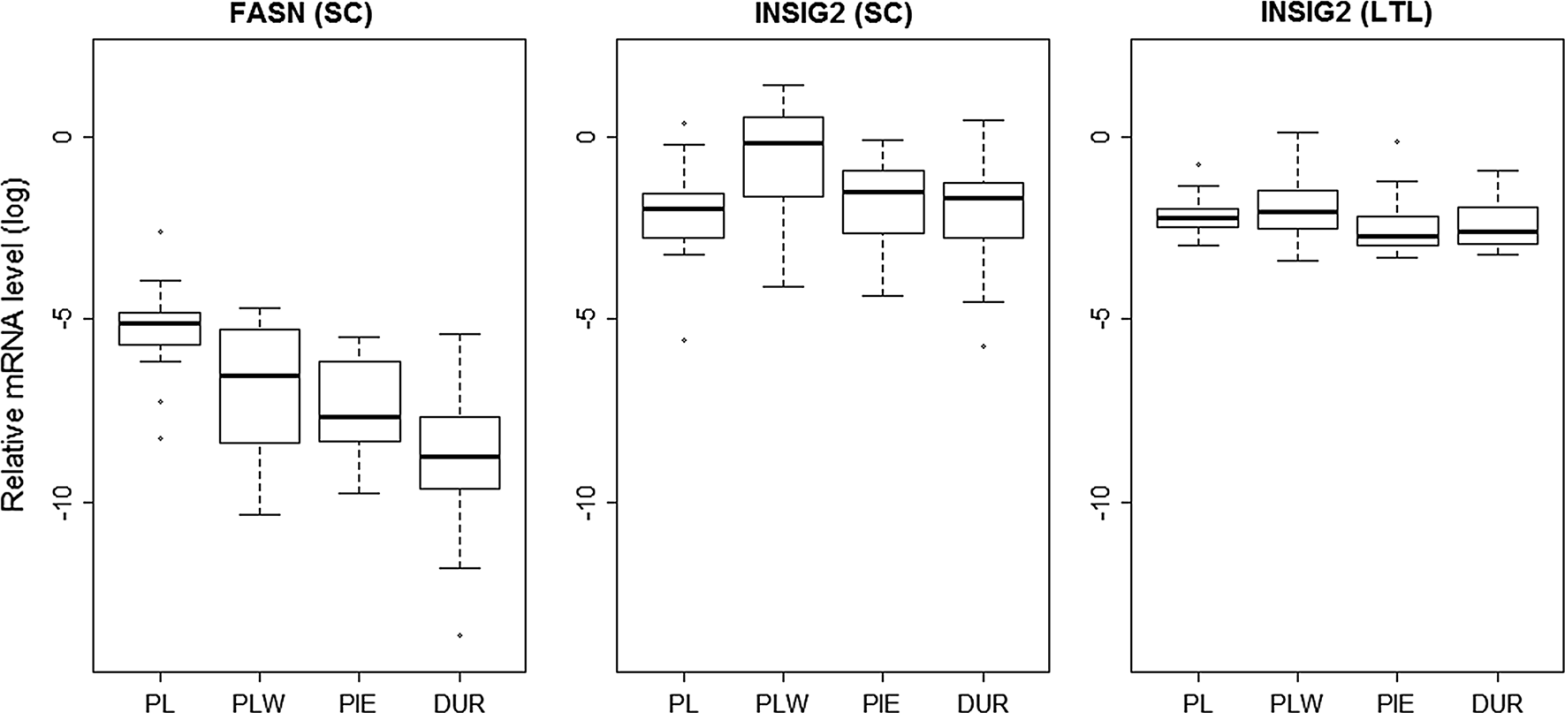
The mean abundance of *FASN* and *INSIG2* transcript levels in subcutaneous fat tissue (SC) and the *longissimus thoracis et lumborum* (LTL) muscle. *PL* Polish Landrace, *PLW* Polish Large White, *PIE* Pietrain, *DUR* Duroc

The mean abundance of *FASN* and *INSIG2* transcript levels was examined in the *longissimus thoracis et lumborum* (LTL) muscle and SC tissue. In general, the level of *FASN* mRNA in SC was the highest in PL and the lowest in Duroc pigs (Fig. 2). A similar relationship was observed also for other genes involved in lipid metabolism, i.e. *ME*, *SCD* and *ACACA*, which transcript levels were elevated in the SC of PL pigs when compared to other analyzed breeds [17–19]. Interestingly, Miao et al. [20] claimed that the *FASN* transcript was more abundant in SC of Jinhua pigs (a fat Chinese breed) than in the Landrace pigs (a leaner breed). Furthermore, considering that gilts in our study were kept under identical conditions and slaughtered at the same body weight, one may conclude of a significant genetic impact on observed differences between breeds. We detected no *FASN* transcripts in LTL and we assumed that it was due to the lack or a very low level of its expression in this tissue. A similar result was reported by Ding et al. [21], who also did not detect the *FASN* transcripts in the muscle of Newsham and Duroc pigs. It should be mentioned, that in pigs FAs are synthesized mainly in adipose tissue [22]. However, Yu et al. [23] showed that the *FASN* transcript was present in LTL of Lantang and Landrace pigs. This discrepancy may be caused by changes in transcript levels during postnatal development, as it was suggested by Duran-Montgé et al. [24]. The authors showed that the expression of some genes related to lipid metabolism, including *FASN*, decline with animal weight and in pigs at a 100-kg body weight the *FASN* mRNA abundance in muscle was only 1/4 of the transcript level of pigs at 60 kg.

We also analyzed a relationship between c.-2335C>T polymorphism, occurring in a consensus sequence for the SP1 transcription factor, and the transcript level of *FASN* in Duroc pigs. The abundance of the transcript in SC was higher (p = 0.041) in CT pigs when compared to the CC ones. Unfortunately, due to an uneven distribution of the variants we could not test the effect of the TT genotype. Nonetheless this result is surprising, since this polymorphism disrupts a putative binding site for the SP1 transcription factor, so one could assume that the effect would be opposite, e.g. lower transcript level. It can be speculated that other mechanisms may affect the transcript level, e.g. methylation pattern, since this SNP occurs within a predicted CpG island.

With regard to the *INSIG2* gene we observed breed- and tissue-specific transcript level variability, as well as a positive correlation between the *INSIG2* mRNA levels in SC and LTL tissues (r = 0.50, p < 0.0001). In PLW the mRNA abundance in SC was significantly higher (p < 0.01) than in LTL. For other breeds this tendency was similar, although not statistically significant. Moreover, the *INSIG2* transcript level in SC of PLW was higher in comparison to PL, Duroc and Pietrain (Fig. 2). Also results obtained by Yingkai et al. [25] indicated a 1.3× higher level of the *INSIG2* transcript in adipose tissue in fat Rongchang pigs than in leaner Landrace. Thus it could be expected, that we should observe the lowest *INSIG2* transcript level in the leanest (in terms of BFT and AF (abdominal fat) values) breed among included in our study, i.e. in Pietrain [9]. However no such correlation was detected, what may suggest, that *INSIG2* transcript level depends also on other factors than breed (lean or fat) and tissue type.

It should also be stressed that we found a negative correlation (r = −0.25, p = 0.032) between transcript levels of *FASN* and *INSIG2* in SC. This result is consistent with the findings of Takaishi et al. [7], who reported that the *INSIG2* overexpression leads to a reduction in mRNA level of *ACC*, *FASN*, *SCD* and *GPAT*. It is also in agreement with INSIG2 biological function, which is regulation of cholesterol homeostasis through binding/releasing mechanism of SCAP/SREBP complex in the endoplasmic reticulum, and thus blocking or enabling SREBP proteolytic activation and action as a factor regulating among others *FASN* gene transcription [6].

### *FASN* and *INSIG2* polymorphisms and transcript levels in relation to fatty acid composition

The importance of meat FAs profile stems mainly from two reasons. On the one hand meat, as a substantial source of FA in the diet, needs to answer consumers requirements of healthy meat, i.e. higher PUFA to SFA ratio and more optimal balance of n-6 to n-3 PUFA [2]. On the other hand, FA composition influences significantly firmness and softness of the meat fat, determining its processing.

Since both studied genes encode proteins playing crucial roles in lipid metabolism, we analyzed an association of their transcript level and polymorphisms with FA composition. At first, we found that the *FASN* gene transcript level in SC was negatively correlated with the C20:2 concentration (r = −0.24, p = 0.048), while with regard to *INSIG2* the correlation concerned C18:2 in SC (r = 0.23, p = 0.046) and MUFA (r = 0.33, p = 0.003), C16:1 (r = 0.24, p = 0.035) and C18:1 n9 (r = 0.35, p = 0.002) in LTL. Furthermore, two *FASN* (c.-2335C>T and c.*264A>G) and one *INSIG2* (c.-5527C>G) polymorphisms showed several breed- and tissue-specific associations with FA composition in SC and LTL tissues. The *INSIG2* polymorphism was associated with the SFA content in LTL tissue of the PL breed and MUFA content in LTL tissue of the PLW breed, while the *FASN* polymorphism (c.-2335C>T) was associated with PUFA content in SC tissue of the Pietrain breed. The increase of FA unsaturation, however desirable from consumer point of view, may lead to technological difficulties in meat processing, in contrast to higher percentage of SFA, which is more favorable during meat transformation [26]. Associations were also observed for specific FA in different breeds and tissues (Table 2). However, due to a limited number of animals, these results should be considered as preliminary suggestions.

**Table 2:** Association of three polymorphisms in the *FASN* and *INSIG2* genes with fatty acid composition

FA	Gene	Breed	Tissue	Mean value of FA ± SE (n)		
				11^a^	12^b^	22^c^
SFA	*INSIG2* ^1^	PL	LTL	40.284 ± 0.568 (7)	40.823^a^ ± 0.323 (11)	42.798^a^ ± 1.054 (3)
MUFA	*INSIG2* ^1^	PLW	LTL	49.149^a^ ± 0.507 (14)	46.682^a^ ± 1.017 (6)	–
PUFA	*FASN* ^2^	Pietrain	SC	20.014^ab^ ± 0.138 (3)	17.706^a^ ± 0.515 (6)	17.034^b^ ± 0.746 (4)
C14:0	*FASN* ^3^	Duroc	LTL	1.367^b^ ± 0.035 (5)	1.447^a^ ± 0.030 (11)	1.589^ab^ ± 0.066 (4)
C17:0	*INSIG2* ^1^	PLW	LTL	0.293^a^ ± 0.018 (15)	0.351^a^ ± 0.028 (7)	–
C20:0	*INSIG2* ^1^	PL	LTL	0.266^B^ ± 0.028 (7)	0.333^a^ ± 0.031 (11)	0.532^aB^ ± 0.104 (3)
C16:1	*FASN* ^3^	Duroc	SC	1.633 ± 0.084 (5)	1.755^A^ ± 0.057 (11)	1.432^A^ ± 0.083 (5)
C18:1 n9	*INSIG2* ^1^	PLW	LTL	41.608^a^ ± 0.444 (15)	39.489^a^ ± 1.155 (7)	–
C20:1	*FASN* ^2^	Pietrain	SC	0.754^ab^ ± 0.06 (4)	0.912^a^ ± 0.036 (6)	0.960^b^ ± 0.056 (4)
C18:2	*INSIG2* ^1^	PL	SC	13.128^ab^ ± 0.519 (8)	11.638^a^ ± 0.390 (11)	10.578^b^ ± 0.712 (3)
C20:2	*FASN* ^2^	PL	LTL	0.459^a^ ± 0.028 (9)	0.354^ab^ ± 0.018 (5)	0.501^b^ ± 0.064 (4)
C18:3	*FASN* ^3^	PL	SC	2.949^B^ ± 0.220 (7)	3.023^A^ ± 0.177 (6)	2.311^AB^ ± 0.080 (5)
	*INSIG2* ^1^	PL	LTL	1.543^ab^ ± 0.210 (7)	2.017^a^ ± 0.117 (9)	2.267^b^ ± 0.100 (3)
cholesterol	*FASN* ^2^	Duroc	LTL	7.304^A^ ± 1.031 (15)	15.169^A^ ± 2.061 (6)	–

^1^ *INSIG2* c.-5527C>G

^2^ *FASN* c.-2335C>T

^3^ *FASN* c.*264A>G

^a^Homozygotes for a wild allele (according to the reference genome sequence)

^b^Heterozygotes

^c^Homozygotes for the detected polymorphic variant

In pigs several associations between *FASN* nucleotide substitutions and FA content were documented. Muñoz et al. [12] found that allele A (c.1254A>G) is positively correlated with the lower C20:1(n-9) content in back fat of the Landrace breed. Kim et al. [16] reported higher levels of C16:1 and C18:1 in Korean native pigs crossed with Yorkshire pigs, with the CC or CT genotype (c.265C>T) and AA or AC (c.6545A>C) in comparison to the TT and CC genotypes, respectively. The effect of c.265C>T polymorphism (exon 4) was also analyzed by Maharani et al. [27], who confirmed a higher proportion of C16:1, C18:1 and MUFA and lower contents of PUFA, including C18:2 and C18:3, in Duroc heterozygotes than in CC homozygotes. Our study also indicates that two polymorphisms (c.-2335C>T and c.*264A>G) of this gene are associated with the FA composition in back fat and *longissimus thoracis et lumborum* tissues.

Associations of *INSIG2* polymorphism with the FA contents were also reported. A higher SFA concentration and a decreased PUFA level in IMF of heterozygotes (c.*121_122delGT polymorphism) of Mangalica x Pietrain pigs was described by Pertek [14]. We observed higher C18:2 level of PL gilts with the CC (c.-5527C>G) genotype. It should be stressed, that C18:2 (linoleic acid) is a main factor of fat tissue firmness [2]. With the increase of C18:2 percentage the lowering of melting point is observed, which leads to worse fat firmness (soft fat), causing technological difficulties in the meat processing: cutting, grinding and slicing [26]. As it was showed by Suzuki et al. [28] the heritability of C18:2 in outer and inner SC tissue is quite high (0.44 and 0.32 respectively), suggesting that FA content is significantly controlled by genetic factors.

## Conclusion

Among 20 polymorphisms identified in regulatory sequences, mainly in 5′-flanking regions, of the *FASN* and *INSIG2* genes 3 SNPs and 1 indel in *FASN* gene (c.-2908G>A, c.-2335C>T, c.*42_43insCCCCA and c.*264A>G) were associated with back fat thickness. Moreover, the transcription profile of the analyzed genes was breed- (for both genes) and tissue-specific (for *INSIG2*), as well as correlated with the content of several FAs. Thus, we conclude that *FASN* is an interesting candidate gene for SC tissue accumulation, while *INSIG2* for the FA profile in the studied tissues.

## Electronic supplementary material

Below is the link to the electronic supplementary material.

Supplementary material 1 (DOC 50 kb)

Supplementary material 2 (DOC 55 kb)
