# Supplementary material for: Pig fatness in relation to FASN and INSIG2 genes polymorphism and their transcript level
Source: Mol Biol Rep. 2016 Mar 10;43:381–9. doi: 10.1007/s11033-016-3969-z (PMC4831998; doi:10.1007/s11033-016-3969-z)
Supplement: Supplementary file 1 — Supplementary material 1 (DOC 50 kb) [file 11033_2016_3969_MOESM1_ESM.doc]

Suppl table 1. Primers used in amplification of *FASN* and *INSIG2* fragments

| **Gene** | **Application** | **PCR primer sequence** | **Annealing temperature**  **(°C)** | **PCR product length**  **(bp)** |
| --- | --- | --- | --- | --- |
| *FASN* | PCR (5'-flanking) | F: TGAGCAGAGCCCCACTGAC | 60.5 | 1094 |
|  |  | R: TTGCTAGGCGATAGGGTGATG |  |  |
|  | PCR (5'-flanking, exon 1) | F: CACCCTATCGCCTAGCAACG | 61.0 | 639 |
|  |  | R: GAGGCAGAGCGAGGATGGA |  |  |
|  | PCR (exon 1, exon 2) | F: CCAGAGAGACGGCAGCG | 62.0 | 314 |
|  |  | R: GTGTTCGCCTGCTTGGAGT |  |  |
|  |  | R: TCCTCCAGGTTCTCCGACT |  |  |
|  | PCR (3'UTR) | F: GTCCATCCTCAGCATCATCC | 57.0 | 461 |
|  |  | R: TAAAAATGAAACGGGGTCCA |  |  |
|  | Real-time PCR | F: ATCGTGAATGCCCTGTGTG  R: GGTTCGGGGTGTGGTAGTG | 65 | 169 |
|  | 5’RACE | OUT: GCCGTGTCTATGGTGATGCT | 58 | - |
|  |  | IN: CTCACACCCACCCAGACAC | 58 | - |
| *INSIG2* | PCR (5'-flanking) | F: GGTTCTTGCTTTTTAGGTCTTG | 62.0 | 601 |
|  |  | R: GAGGTCAGGTTACTGTGTTCTTG |  |  |
|  | PCR (5'-flanking, exon 1) | F: CCTCAAGAACACAGTAACCTGAC | 64.0 | 826 |
|  |  | R: GTCCCTACCCCCAACAACAA |  |  |
|  | PCR (exon 6, 3'UTR) | F: TGAGGAATAAAGGGAGCCAAT | 60.0 | 857 |
|  |  | R: GCTGATAAACTCTCCTGCTCGT |  |  |
|  | PCR (3'UTR) | F: CTCAGATAGCTTTGGGTGTTTT | 58.0 | 665 |
|  |  | R: ACAAACGCAGTGGAATACTGT |  |  |
|  | Real-time PCR | F: TCTTTCCACCGGATGTGATT  R: GTGGTTCTCCTAGATGCCTGTC | 64 | 125 |
| *ACTB* | Real-time PCR | F: CCCCCGACACCAGGGCGTGAT  R: CGGCCAGAGGCGTACAGGGACAG | 63 | 330 |
| *PPIA* | Real-time PCR | F: CACAAACGGTTCCCAGTTTT  R: TGTCCACAGTCAGCAATGGT | 63 | 171 |
