# Supplementary material for: Pig fatness in relation to FASN and INSIG2 genes polymorphism and their transcript level
Source: Mol Biol Rep. 2016 Mar 10;43:381–9. doi: 10.1007/s11033-016-3969-z (PMC4831998; doi:10.1007/s11033-016-3969-z)
Supplement: Supplementary file 2 — Supplementary material 2 (DOC 55 kb) [file 11033_2016_3969_MOESM2_ESM.doc]

Suppl table 2. Frequencies of alleles of polymorphisms identified during preliminary screening

| **Gene** | **Polymorphism** | **Localization** | **Frequency** | | | | | | | |
| --- | --- | --- | --- | --- | --- | --- | --- | --- | --- | --- |
|  |  |  | **Allele 1 (wild type)** | | | | **Allele 2 (polymorphic type)** | | | |
|  |  |  | **PL** | **PLW** | **Pietrain** | **Duroc** | **PL** | **PLW** | **Pietrain** | **Duroc** |
| *FASN* | c.-3220C>T | 5’-flanking | 0,44 | 0,62 | 0,32 | 0,36 | 0,56 | 0,38 | 0,68 | 0,64 |
|  | c.-3023C>T | 5’-flanking | 0,84 | 0,71 | 0,89 | 1,00 | 0,16 | 0,29 | 0,11 | 0,00 |
|  | c.-2943A>C | 5’-flanking | 0,63 | 0,39 | 0,83 | 0,93 | 0,37 | 0,61 | 0,17 | 0,07 |
|  | c.-2908G>A | 5’-flanking | 0,63 | 0,39 | 0,83 | 0,98 | 0,37 | 0,61 | 0,17 | 0,02 |
|  | c.-2863G>A | 5’-flanking | 0,08 | 0,02 | 0,13 | 0,36 | 0,92 | 0,98 | 0,87 | 0,64 |
|  | c.-2649T>C | 5’-flanking | 0,63 | 0,39 | 0,83 | 0,93 | 0,37 | 0,61 | 0,17 | 0,07 |
|  | c.-2631G>T | 5’-flanking | 0,08 | 0,02 | 0,17 | 0,36 | 0,92 | 0,98 | 0,83 | 0,64 |
|  | c.-2392T>G | 5’-flanking | 0,56 | 0,20 | 0,50 | 0,50 | 0,44 | 0,80 | 0,50 | 0,50 |
|  | c.-2335C>T | 5’-flanking | 0,67 | 0,93 | 0,53 | 0,85 | 0,33 | 0,07 | 0,47 | 0,15 |
|  | c.-2333_2334insG | 5’-flanking | 0,44 | 0,80 | 0,50 | 0,50 | 0,56 | 0,20 | 0,50 | 0,50 |
|  | c.*42_43insCCCCA | 3’UTR | 1,00 | 0,83 | 1,00 | 1,00 | 0,00 | 0,17 | 0,00 | 0,00 |
|  | c.*264A>G | 3’UTR | 0,55 | 0,39 | 0,65 | 0,52 | 0,45 | 0,61 | 0,35 | 0,48 |
| *INSIG2* | c.-5616G>T | 5’-flanking | 1,00 | 1,00 | 1,00 | 0,80 | 0,00 | 0,00 | 0,00 | 0,20 |
|  | c.-5603T>C | 5’-flanking | 1,00 | 1,00 | 1,00 | 0,80 | 0,00 | 0,00 | 0,00 | 0,20 |
|  | c.-5527C>G | 5’-flanking | 0,81 | 0,83 | 1,00 | 0,83 | 0,19 | 0,17 | 0,00 | 0,17 |
|  | c.-5271G>A | 5’UTR | 1,00 | 1,00 | 1,00 | 0,80 | 0,00 | 0,00 | 0,00 | 0,20 |
|  | c.*423G>A | 3’UTR | 0,89 | 0,72 | 0,76 | 0,83 | 0,11 | 0,28 | 0,24 | 0,17 |
|  | c.*463C>T | 3’UTR | 0,91 | 0,97 | 1,00 | 1,00 | 0,09 | 0,03 | 0,00 | 0,00 |
|  | c.*725T>C | 3’UTR | 0,83 | 0,92 | 1,00 | 0,64 | 0,17 | 0,08 | 0,00 | 0,36 |
|  | c.*793A>C | 3’UTR | 0,83 | 0,92 | 1,00 | 0,50 | 0,17 | 0,08 | 0,00 | 0,50 |
